# Supplementary material for: Modeling glioblastoma heterogeneity as a dynamic network of cell states
Source: Mol Syst Biol. 2021 Sep 16;17(9):e10105. doi: 10.15252/msb.202010105 (PMC8444284; doi:10.15252/msb.202010105)
Supplement: Supplementary file 6 — Source Data for Figure 5 [file MSB-17-e10105-s004.zip › Figure5A_sourcedata/GSEA_3071/hallmarks_stateIV.GseaPreranked.1623742551702/gsea_report_for_na_neg_1623742551702.html]

Report for na\_neg 1623742551702 [GSEA]

| GS  follow link to MSigDB | GS DETAILS | SIZE | ES | NES | NOM p-val | FDR q-val | FWER p-val | RANK AT MAX | LEADING EDGE || 1 | HALLMARK\_EPITHELIAL\_MESENCHYMAL\_TRANSITION | Details ... | 132 | -0.61 | -3.38 | 0.000 | 0.000 | 0.000 | 629 | tags=49%, list=12%, signal=54% |
| 2 | HALLMARK\_E2F\_TARGETS | Details ... | 147 | -0.56 | -3.14 | 0.000 | 0.000 | 0.000 | 1340 | tags=64%, list=25%, signal=83% |
| 3 | HALLMARK\_G2M\_CHECKPOINT | Details ... | 128 | -0.54 | -2.99 | 0.000 | 0.000 | 0.000 | 1046 | tags=59%, list=20%, signal=71% |
| 4 | HALLMARK\_MYC\_TARGETS\_V1 | Details ... | 129 | -0.47 | -2.57 | 0.000 | 0.000 | 0.000 | 1971 | tags=74%, list=37%, signal=115% |
| 5 | HALLMARK\_IL2\_STAT5\_SIGNALING | Details ... | 87 | -0.47 | -2.36 | 0.000 | 0.000 | 0.000 | 878 | tags=44%, list=17%, signal=52% |
| 6 | HALLMARK\_UV\_RESPONSE\_DN | Details ... | 82 | -0.47 | -2.33 | 0.000 | 0.000 | 0.000 | 742 | tags=39%, list=14%, signal=45% |
| 7 | HALLMARK\_MYC\_TARGETS\_V2 | Details ... | 35 | -0.53 | -2.19 | 0.000 | 0.000 | 0.000 | 2051 | tags=86%, list=39%, signal=139% |
| 8 | HALLMARK\_COAGULATION | Details ... | 41 | -0.51 | -2.15 | 0.000 | 0.000 | 0.001 | 454 | tags=39%, list=9%, signal=42% |
| 9 | HALLMARK\_ANGIOGENESIS | Details ... | 21 | -0.60 | -2.15 | 0.000 | 0.000 | 0.002 | 325 | tags=43%, list=6%, signal=45% |
| 10 | HALLMARK\_MTORC1\_SIGNALING | Details ... | 120 | -0.39 | -2.13 | 0.000 | 0.000 | 0.002 | 1272 | tags=48%, list=24%, signal=62% |
| 11 | HALLMARK\_INFLAMMATORY\_RESPONSE | Details ... | 66 | -0.44 | -2.10 | 0.000 | 0.000 | 0.003 | 741 | tags=39%, list=14%, signal=45% |
| 12 | HALLMARK\_HYPOXIA | Details ... | 105 | -0.38 | -2.05 | 0.000 | 0.001 | 0.008 | 380 | tags=27%, list=7%, signal=28% |
| 13 | HALLMARK\_REACTIVE\_OXYGEN\_SPECIES\_PATHWAY | Details ... | 26 | -0.54 | -2.04 | 0.002 | 0.001 | 0.011 | 942 | tags=54%, list=18%, signal=65% |
| 14 | HALLMARK\_MITOTIC\_SPINDLE | Details ... | 106 | -0.38 | -2.02 | 0.000 | 0.002 | 0.014 | 1474 | tags=52%, list=28%, signal=70% |
| 15 | HALLMARK\_APICAL\_JUNCTION | Details ... | 88 | -0.39 | -2.00 | 0.000 | 0.002 | 0.017 | 828 | tags=34%, list=16%, signal=40% |
| 16 | HALLMARK\_UNFOLDED\_PROTEIN\_RESPONSE | Details ... | 55 | -0.43 | -1.99 | 0.000 | 0.002 | 0.018 | 1825 | tags=62%, list=34%, signal=93% |
| 17 | HALLMARK\_ALLOGRAFT\_REJECTION | Details ... | 55 | -0.42 | -1.98 | 0.002 | 0.002 | 0.021 | 1760 | tags=60%, list=33%, signal=89% |
| 18 | HALLMARK\_GLYCOLYSIS | Details ... | 96 | -0.38 | -1.97 | 0.000 | 0.002 | 0.021 | 943 | tags=38%, list=18%, signal=45% |
| 19 | HALLMARK\_MYOGENESIS | Details ... | 74 | -0.40 | -1.96 | 0.000 | 0.002 | 0.022 | 252 | tags=20%, list=5%, signal=21% |
| 20 | HALLMARK\_TNFA\_SIGNALING\_VIA\_NFKB | Details ... | 103 | -0.35 | -1.88 | 0.000 | 0.005 | 0.061 | 686 | tags=30%, list=13%, signal=34% |
| 21 | HALLMARK\_INTERFERON\_GAMMA\_RESPONSE |  | 76 | -0.36 | -1.79 | 0.002 | 0.010 | 0.136 | 1187 | tags=39%, list=22%, signal=50% |
| 22 | HALLMARK\_P53\_PATHWAY |  | 91 | -0.35 | -1.76 | 0.002 | 0.011 | 0.154 | 919 | tags=33%, list=17%, signal=39% |
| 23 | HALLMARK\_DNA\_REPAIR |  | 74 | -0.36 | -1.74 | 0.004 | 0.013 | 0.176 | 1361 | tags=47%, list=26%, signal=63% |
| 24 | HALLMARK\_APOPTOSIS |  | 79 | -0.34 | -1.71 | 0.002 | 0.015 | 0.212 | 599 | tags=25%, list=11%, signal=28% |
| 25 | HALLMARK\_ESTROGEN\_RESPONSE\_LATE |  | 82 | -0.33 | -1.70 | 0.000 | 0.015 | 0.221 | 395 | tags=22%, list=7%, signal=23% |
| 26 | HALLMARK\_COMPLEMENT |  | 76 | -0.34 | -1.67 | 0.009 | 0.018 | 0.269 | 812 | tags=32%, list=15%, signal=37% |
| 27 | HALLMARK\_ADIPOGENESIS |  | 77 | -0.30 | -1.50 | 0.014 | 0.060 | 0.670 | 615 | tags=19%, list=12%, signal=22% |
| 28 | HALLMARK\_ESTROGEN\_RESPONSE\_EARLY |  | 86 | -0.27 | -1.38 | 0.057 | 0.123 | 0.914 | 796 | tags=24%, list=15%, signal=28% |
| 29 | HALLMARK\_ANDROGEN\_RESPONSE |  | 50 | -0.30 | -1.37 | 0.086 | 0.125 | 0.922 | 981 | tags=34%, list=19%, signal=41% |
| 30 | HALLMARK\_WNT\_BETA\_CATENIN\_SIGNALING |  | 18 | -0.39 | -1.35 | 0.133 | 0.134 | 0.935 | 566 | tags=28%, list=11%, signal=31% |
| 31 | HALLMARK\_HEDGEHOG\_SIGNALING |  | 24 | -0.35 | -1.32 | 0.143 | 0.156 | 0.965 | 368 | tags=29%, list=7%, signal=31% |
| 32 | HALLMARK\_XENOBIOTIC\_METABOLISM |  | 65 | -0.27 | -1.29 | 0.124 | 0.173 | 0.986 | 1020 | tags=31%, list=19%, signal=38% |
| 33 | HALLMARK\_CHOLESTEROL\_HOMEOSTASIS |  | 35 | -0.31 | -1.28 | 0.154 | 0.178 | 0.989 | 983 | tags=37%, list=19%, signal=45% |
| 34 | HALLMARK\_INTERFERON\_ALPHA\_RESPONSE |  | 39 | -0.28 | -1.19 | 0.254 | 0.271 | 1.000 | 1187 | tags=33%, list=22%, signal=43% |
| 35 | HALLMARK\_PI3K\_AKT\_MTOR\_SIGNALING |  | 52 | -0.26 | -1.18 | 0.246 | 0.282 | 1.000 | 744 | tags=21%, list=14%, signal=24% |
| 36 | HALLMARK\_FATTY\_ACID\_METABOLISM |  | 53 | -0.23 | -1.05 | 0.396 | 0.472 | 1.000 | 1703 | tags=47%, list=32%, signal=69% |
| 37 | HALLMARK\_SPERMATOGENESIS |  | 36 | -0.25 | -1.04 | 0.399 | 0.467 | 1.000 | 1382 | tags=44%, list=26%, signal=60% |
| 38 | HALLMARK\_BILE\_ACID\_METABOLISM |  | 25 | -0.26 | -0.97 | 0.502 | 0.577 | 1.000 | 297 | tags=16%, list=6%, signal=17% |
| 39 | HALLMARK\_APICAL\_SURFACE |  | 18 | -0.28 | -0.93 | 0.529 | 0.629 | 1.000 | 705 | tags=28%, list=13%, signal=32% |
| 40 | HALLMARK\_PROTEIN\_SECRETION |  | 45 | -0.21 | -0.91 | 0.583 | 0.654 | 1.000 | 111 | tags=7%, list=2%, signal=7% |
| 41 | HALLMARK\_PEROXISOME |  | 32 | -0.21 | -0.87 | 0.669 | 0.701 | 1.000 | 847 | tags=25%, list=16%, signal=30% |
| 42 | HALLMARK\_IL6\_JAK\_STAT3\_SIGNALING |  | 28 | -0.19 | -0.75 | 0.791 | 0.857 | 1.000 | 511 | tags=14%, list=10%, signal=16% |
| 43 | HALLMARK\_UV\_RESPONSE\_UP |  | 70 | -0.15 | -0.72 | 0.895 | 0.876 | 1.000 | 462 | tags=13%, list=9%, signal=14% |
Table: Gene sets enriched in phenotype **na **[plain text format]****
